# AGE BIAS IN PEOPLE’S DAILY ACROSS 72 YEARS

**DOI:** 10.1093/geroni/igae098.3264

**Published:** 2024-12-31

**Authors:** Chunyan Mai, Amber X Chen, Ji Cao, Helene H Fung

**Affiliations:** The Chinese University of Hong Kong, Hong Kong, China (People’s Republic); University of California Santa Barbara, Santa Barbara, California, United States; The Chinese University of Hong Kong, Hong Kong, China (People’s Republic); The Chinese University of Hong Kong, Hong Kong, China (People’s Republic)

## Abstract

**Background:**

Older adults were traditionally portrayed to be respected in China, a collectivistic and culturally tight society. However, findings are mixed on attitudes towards the older adults in contemporary China, amidst socio-economic transformation and cultural shifts. Addressing this gap, our study investigates the evolution of societal attitudes towards the older group, as reflected in narratives from the People’s Daily Newspaper (Renmin Ribao) from 1950 to 2021.

**Methods:**

Societal attitudes were measured with three metrics: moral judgment (virtue versus vice), ageist evaluations (positive versus negative), and stereotypes (warmth versus competence), utilizing natural language processing. Age biases (Virtue Bias, etc.) refer to the association between attitude-related words (virtue, etc.) and the older group relative to the younger group. To explain the overtime change in the Age Biases, time series models and Granger tests were applied to examine the correlation and precedence between cultural values and age biases.

**Results:**

The results of time series models revealed that after accounting for GDP per capita, collectivistic values were positively correlated with Warmth Bias. Additionally, cultural looseness exhibited positive correlations with Virtue Bias, Positive Ageist Bias, and Warmth Bias. Granger tests revealed that declines in cultural looseness preceded declining Virtue Bias and Positive Ageist Bias.

**Discussion:**

This is the first known study longitudinally exploring societal attitudes towards the older group in China. Our findings highlight the significance of the temporal dynamics of societal attitudes along with cultural changes.

